# Efficacy of behavioral classroom programs in primary school. A meta-analysis focusing on randomized controlled trials

**DOI:** 10.1371/journal.pone.0201779

**Published:** 2018-10-10

**Authors:** Betty Veenman, Marjolein Luman, Jaap Oosterlaan

**Affiliations:** Clinical Neuropsychology section, Vrije Universiteit Amsterdam, Amsterdam, The Netherlands; University of New South Wales, AUSTRALIA

## Abstract

**Objective:**

This meta-analysis evaluated the efficacy of behavioral classroom programs on symptoms of Attention-deficit Hyperactivity Disorder or Oppositional Defiant and/or Conduct Disorder in primary school children.

**Method:**

Online database searches (in PubMed, Embase, Psycinfo, and Eric) yielded nineteen randomized controlled trials (*N* = 18,094), comparing behavioral classroom programs (including multimodal programs involving a classroom program) to no treatment/treatment as usual. Random-effects meta-analyses were conducted for teacher-rated and classroom-observed disruptive classroom behavior and for classroom-observed on-task behavior. Post-hoc analyses investigated whether effects depended on type and severity of problem behavior. Meta-regressions studied the moderating effects of age, gender, and intervention duration.

**Results:**

Small positive effects were found on teacher-rated disruptive behavior (*d* = -0.20) and classroom-observed on-task behavior (*d* = 0.39). Program effects on teacher-rated disruptive behavior were unrelated to age, gender, type and severity, but negatively associated with intervention duration (*R*^2^ = 0.43).

**Conclusion:**

Behavioral classroom programs have small beneficial effects on disruptive behavior and on-task behavior. Results advocate universal programs for entire classrooms to prevent and reduce disruptive classroom behavior.

## Introduction

Attention-Deficit Hyperactivity Disorder (ADHD) and Disruptive behavior disorders are two of the most common psychiatric disorders in children [1, 2] and comorbidity between these disorders is very substantial [3]. The main characteristics of ADHD are hyperactivity-impulsivity and inattention [4]. Disruptive Behavior Disorders can be subdivided into Oppositional Defiant Disorder (ODD) and Conduct Disorder (CD). Main characteristics of ODD and CD are negativism, hostility, and disobedience towards authority figures, while aggression and serious violation of the basic rights of others are essential to CD [4]. Children with externalizing behavior problems often display disruptive behavior in school such as talking aloud, disobedience and off-task behavior [5]. Moreover, these children have a high risk of academic underachievement and school dropout [6, 7]. Externalizing behavior problems are further associated with peer problems and family dysfunction as well as adverse events later in life such as work-related problems, substance abuse and antisocial behavior [8, 9]. Given the impairments and adverse outcomes associated with disruptive behavior, interventions at an early age are crucial to prevent escalation of problem behavior later in life.

Medication is commonly used to effectively reduce the core symptoms of ADHD and to reduce severe and persistent aggression. However, 20–30% of children with ADHD using psychostimulants fail to show symptom improvements or suffer from adverse side effects such as insomnia, headache or loss of appetite [10, 11] and the evidence for long-term effects of stimulant medication is limited [12]. Besides that, medication is discouraged as treatment for ODD and CD, except for cases showing severe aggression [10]. Therefore, non-pharmacological interventions are needed that effectively reduce disruptive behavior. A meta-analysis comparing effects of behavioral and non-behavioral interventions (all predominantly family-based), showed highest effect sizes for behavioral therapy on externalizing behavior problems such as ADHD, ODD and CD [13]. As children with externalizing behavior problems frequently display disruptive behavior at school [5] and schools have the advantage of reaching nearly all children, school-based behavioral interventions may provide a very important lead to the treatment of disruptive behavior.

Behavioral programs are often used at school to address disruptive classroom behavior. The majority of these programs use antecedent techniques to prevent behavioral problems (e.g. by clear rules and effective instructions) and consequent techniques to reinforce appropriate behavior and reduce inappropriate behavior (e.g. by reward systems and time-out systems). The Summer Treatment Program, for example, contains a behavioral classroom program for children with ADHD, in which positive reinforcement is used within classroom-settings and during activities outside the classroom such as during sports [14]. The Good Behavior Game is another example of a widely-used group behavioral program, in which small groups of students are rewarded for the behavior of one or more students in their group [15].

Several reviews and meta-analyses have addressed the efficacy of behavioral classroom programs on disruptive behavior, and on ADHD symptoms in particular [5, 15–28]. Unfortunately, none of the available reviews and meta-analyses focused specifically on classroom-based behavioral programs and none focused exclusively on randomized controlled trials (RCTs), which are regarded as the gold standard due to a minimization of biases and possibly confounding factors [29–31]. Therefore, it is difficult to draw firm conclusions regarding the effects of behavioral classroom programs on disruptive behavior within the classroom and the potential moderators influencing these effects. The meta-analysis of Fabiano and colleagues [28], for example, investigated the effectiveness of behavioral treatments for children with ADHD and reported behavioral programs to be highly effective (unweighted effects being .83 in between-group studies, .70 in pre-post studies, 2.64 in within-group studies, and 3.78 in single subject studies). The large difference reported in that meta-analysis between the effect size for single subject designs (3.78) compared to that for between-group studies (.83) demonstrates how study design can result in inflated effect sizes and thus highlights the importance of RCTs. Unfortunately, only 20 of the 174 studies (11%) included in the meta-analysis of Fabiano and colleagues [28] involved between-group designs and it is unclear if any of those studies were RCT’s. Besides that, only one fourth of the twenty between-group studies involved classroom programs and effects are not reported for classroom behavioral program separately. The meta-analysis of Maggin and colleagues [21] did investigate the effects of classroom programs, but included only group contingency programs (i.e. programs in which groups of students receive a reward based on the behavior of one or more students of that group) instead of focusing on all types of classroom behavior programs. Another recent meta-analysis [27] has investigated the effects of all types of behavioral classroom programs on disruptive classroom behavior for children with symptoms of ADHD. Results of that meta-analysis showed reductions of off-task behavior and of disruptive classroom behavior (standardized mean differences being 0.92 for within-subjects designs and 3.08 for single subject designs), but that meta-analysis did not include any studies using between-subjects designs and thus no RCTs either. Hence, based on the available literature, it is difficult to draw firm conclusions regarding the specific effects (and effect sizes) of behavioral classroom programs on disruptive behavior within the classroom.

Our study contributes to the literature with a comprehensive quantitative meta-analysis of all available RCTs into the effects of behavioral classroom programs in primary school, including multimodal programs that involve a behavioral classroom program. Our focus was on disruptive behavior in the classroom, as assessed by teachers and classroom observations, and on classroom-observed on-task behavior. Based on earlier work, significant intervention effects were expected for disruptive behavior rated by teachers as well as independent classroom observations [32]. However, for the classroom-observed outcomes effects might be smaller as teachers are directly involved in the delivery of treatment, in contrast to the classroom observers (see [33] for a review on this issue). It was also assessed whether effects of behavioral programs depended on the type of problem behavior (ADHD and ODD/CD symptoms) and on severity of problem behavior (by comparing clinical, at-risk, and community samples). Symptoms of ODD and CD were taken together because ODD and CD show resemblance in terms of phenotypical manifestation, high comorbidity, substantial overlap in risk factors associated with both disorders, and similarities in terms of effective treatments [34–36]. Previous studies have suggested better response to behavioral programs for oppositional problems compared to ADHD symptoms and for more severe problem behavior compared to less severe problem behavior [13, 32, 37]. Therefore, larger effects were expected for ODD/CD symptoms than for ADHD symptoms and for samples with more severe problem behavior (clinical and/or at-risk samples compared to community samples). The potential moderating effects of gender, age and intervention duration on outcome were assessed in order to identify for whom and under which conditions behavioral programs would be most effective. The evidence for possible moderating effects of gender is inconclusive as some studies report no moderating effects of gender [38, 39], while others reveal superior treatment effects for boys [40, 41]. The moderating effect of age is inconclusive as meta-analyses on the efficacy of psychosocial interventions reveal inconsistent results [13, 37]. With regard to intervention duration, a positive association was expected with treatment efficacy, since more lengthy programs may result in better treatment response (see [42] but see also [43]).

## Method

This meta-analysis was performed in conformity with the guidelines provided by the PRISMA group (Preferred Reporting Items for Systematic Reviews and Meta-Analysis; see S1 Supplement for the PRISMA Checklist [44]).

### Study selection and description

This meta-analysis included efficacy studies on behavioral classroom programs that met the following inclusion criteria: the study (1) was a randomized controlled trial assessing the effects of behavioral classroom programs compared to ‘no care’ or ‘care as usual’. Behavioral programs were defined as programs using behavioral techniques on a daily basis (e.g. token economy). To incorporate all available RCTs on the efficacy of behavioral classroom programs in this meta-analysis, more comprehensive treatment programs (e.g. multimodal programs or those involving additional cognitive behavioral elements) that used a behavioral classroom program as one of the main elements were also included; (2) included one of the following outcome measures: (a) disruptive classroom behavior (i.e. symptoms of ADHD, ODD/CD, or a combination of those symptoms) as assessed by validated teacher ratings or classroom observations by an independent rater, or (b) classroom-observed on-task behavior. Studies that only assessed disruptive behavior at the classroom level (e.g. total of discipline referrals in the entire classroom) rather than disruptive behavior of individual children were not included; (3) included predominantly elementary school children as participants (6–12 years old on average) in regular education or school-related settings, regardless of severity of problem behavior (thus including community samples, children at risk for and children with clinical externalizing behavior disorders) and (4) were published between 1980 and 1^st^ of July 2016 in an English-language peer-reviewed journal, which is in accordance with the emergence of the third version of the Diagnostic and Statistical Manual of Mental Disorders (DSM-III) [45]. Studies were excluded if: (1) the study focused on children with psychiatric or neurological problems other than ADHD or ODD/CD (e.g. autism or epilepsy); (2) the study solely focused on the enhancement of concentration problems or only on disruptive behavior outside the classroom, such as playground aggression or bullying, since such studies did not target disruptive classroom behavior (such as [46, 47]) or (3) if there were insufficient data to calculate effect sizes (e.g. if only mean and sample size were available, but no standard deviation or standard error). Despite our attempt to obtain the missing data necessary for effect size calculation, two studies were excluded based on this third exclusion criterion [48, 49]. If multiple articles were published using the same sample, we selected the most comprehensive report on that study with the largest sample size or the most encompassing assessment of disruptive classroom behavior. Characteristics of the individual studies included in this meta-analysis (including dependent variables and test statistics) are depicted in Table 1.

**Table 1:** Randomized controlled trials on the effectiveness of classroom-based programs on disruptive and off-task behavior.

Randomization level	Sample inclusion and exclusion criteria	Age range / grade range at T_0_	Intervention duration and elements of treatment and control arm	Parameters used for effect size calculation and other relevant statistical information	Outcome measures and instruments used for effect size calculation	Study quality^a^
Study *1*. *Positive Attitudes toward Learning in School (PALS)* [52]						
Randomization at classroom level	*N* = 90 48 boys; 42 girls Clinical EBD score based on EBD rating scale No exclusion criteria	Kindergarten—Grade 4	1 year Treatment arm: mainly BP, but also CB, social skills and peer tutoring; multimodal: also parent component Control arm: care as usual	Parameters: *r* and *N* for T_0_ (baseline) and T_1_ (1 year), controlling for pretest scores Baseline differences: none Special remarks: the data of cohort 1 was used since only this cohort received BP. The statistic *r* corresponded to the intensity of the school intervention and was based on the number of times advise was given and its duration	Outcome measures: DBP: CRS (T) ADHD: n.a. ODD/CD: n.a. On-task behavior: n.a.	1
Study *2*. *Links to Learning* [53].						
Randomization at school level. Schools were of similar size and in similar proximity to participating mental health services	*N* = 171 124 boys; 47 girls Clinical EBD score based on EBD rating scale (T or P) No exclusion criteria	Kindergarten—Grade 4	3 years Treatment arm: BP, peer tutoring, and Good News Notes (positive feedback to parents): also parent component (but only for 8 weeks) Control arm: care as usual	Parameters: *M*, *SD* and *N* for T_0_ (baseline) and T_1_ (6 months) Baseline differences: off-task: intervention > control Special remarks: none	Outcome measures: DBP: SDQ (T) ADHD: n.a. ODD/CD: n.a. On-task behavior: BOSS Engagement	3
Randomization level	Sample inclusion and exclusion criteria	Age range / grade range at T_0_	Intervention duration and elements of treatment and control arm	Parameters used for effect size calculation and other relevant statistical information	Outcome measures and instruments used for effect size calculation	Study quality^a^
Study *3*. *School-Wide Positive Behavioral Intervention and Supports (SWPBIS)* [54].						
Randomization at school level, with schools being matched at baseline demographics (e.g. school enrollment)	*N* = 12,344 6,482 boys; 5,782 girls Community sample No exclusion criteria	Kindergarten—Grade 2	4 years Treatment arm: BP (schoolwide = universal program) Control arm: waitlist group not receiving intervention during assessment period	Parameters: *t* and *N* for T_0_ (baseline) and T_5_ (4 years), controlling for several school-level and student-level variables (including gender and grade cohort) Baseline differences: none Special remarks: special education status (proxy of severity of problem behavior) did not moderate results	Outcome measures: DBP: TOCA-C, average of ADHD and ODD/CD (T) ADHD: TOCA-C, concentration scale (T) ODD/CD: TOCA-C, aggression scale (T) On-task behavior: n.a.	2
Study *4*. *Multicomponent Competence Enhancement Intervention (MCEI)* [55].						
Randomized block design^b^, with school districts as blocks that were matched on SES, reading achievement and school size	*N* = 309 247 boys; 62 girls At risk for EBD based on teacher and parent rating (CRS-HI) Exclusion criteria: IQ-score < 80, educational placement in program for pervasive developmental disorder or severe EBD	Grade 1–4	2 years Treatment arm: BP, CB and social skills; multimodal: also parent and child component Control arm: information/attention control group of low intensity (max 12 hours per year, but >50% attended no sessions or 2–6 hours)	Parameters: *M*, *SD* and *N* for T_0_ (baseline) and T_1_ (6 months) Baseline differences: SES: control > treatment Special remarks: since *N* differed at pre- and post-assessment, the average *N* was used to calculate the study's effect size	Outcome measures: DBP: BASC, externalizing scale (T) ADHD: CRS (T) ODD/CD: n.a. On-task behavior: n.a.	2
Randomization level	Sample inclusion and exclusion criteria	Age range / grade range at T_0_	Intervention duration and elements of treatment and control arm	Parameters used for effect size calculation and other relevant statistical information	Outcome measures and instruments used for effect size calculation	Study quality^a^
Study *5*. *Check*, *Connect*, *& Expect (CCE)* [56].						
Randomized block design^b^, with schools as blocks that were matched on school size, % of students with IEPs, % students receiving free lunch, and % of Caucasian students	*N* = 207 152 boys; 55 girls At risk for EBD based on teacher rating (SSBD) No exclusion criteria	Grade 1–3	2 years Treatment arm: mainly BP and CB, but if necessary for individual children also social skills, academic tutoring or problem solving with parents Control arm: waitlist group not receiving intervention during assessment period	Parameters: *M*, *SD* and *N* for T_0_ (baseline) and T_1_ (6 months) Baseline differences: SES: control > treatment Special education: control > treatment Special remarks: data of graduates (successfully completing the program) and non-graduates (not having completed the program successfully) were averaged	Outcome measures: DBP: TRF, externalizing scale (T) ADHD: n.a. ODD/CD: n.a. On-task behavior: SSBD, stage 3 (% of AET in 20 minutes)	2
Study *6*. *Good Behavior Game (GBG) or Mastery Learning (ML)* [57].						
Randomized to treatment or external control group at school level, matching on students’ achievements, SES and ethnicity. Within intervention schools, classrooms and new students were randomly assigned to treatment or (internal) control group	*N* = 394 193 boys; 201 girls Community sample in 'at-risk' schools (low SES) No exclusion criteria	Grade 1	1 year 2 treatment arms: GBG group received BP (universal program) and ML group received enriched reading curriculum Control arm: no treatment group. Internal and external control groups were used (control classrooms within intervention school, and separate control schools, respectively)	Parameters: *M*, *SD* and *N* for T_0_ (baseline) and T_1_ (6 months) Baseline differences: none Special remarks: Data of boys and girls were averaged Data of GBG group and external control group were used, to enhance comparability with the other studies in this meta-analysis mostly using external control groups Analyses did not account for severity of problem behavior	Outcome measures: DBP: TOCA-R aggression scale (T) ADHD: n.a. ODD/CD: TOCA-R, aggression scale (T) On-task behavior: n.a.	1
Randomization level	Sample inclusion and exclusion criteria	Age range / grade range at T_0_	Intervention duration and elements of treatment and control arm	Parameters used for effect size calculation and other relevant statistical information	Outcome measures and instruments used for effect size calculation	Study quality^a^
Study *7*. *Daily Report Card* [58].						
Randomization at student level	*N* = 63 54 boys; 9 girls Clinical ADHD based on parent interview (DBD) and on parent and teacher rating (DBD) Exclusion criterion: IQ < 80	6–12 years; Grade 1–6	8 months Treatment arm: BP Control arm: care as usual	Parameters: *M*, *SD* and *N* for T_0_ (baseline) and T_1_ (8 months) Baseline differences: none Special remarks: none	Outcome measures: DBP: DBD (T) and observation (average frequency counts of classroom rule violations) ADHD: DBD, ADHD scale (T) ODD/CD: DBD, average of ODD and CD scale (T) On-task behavior: n.a.	3
Study *8*. *Peacebuilders* [59].						
Randomized block design^b^, with schools as blocks that were matched on geographic proximity, % of ethnic students, % of students receiving free lunch, and % of classrooms with English as a second language	*N* = 4195 Gender ratio n.r. Community sample No exclusion criteria	Kindergarten—Grade 5	1 year Treatment arm: BP and social-emotional skills (universal program) Control arm: waitlist group not receiving intervention during assessment period	Parameters: *t* and *N* for T_0_ (baseline) and T_1_ (6 months), controlling for baseline and gender Baseline differences: n.r. Special remarks: Data were reported separately in this study for Kindergarten—Grade 2 and for Grade 3–5, and were averaged for this meta-analysis For Grade 3–5, program effects were larger for children with more aggression. For Kindergarten—Grade 2, severity of aggression did not moderate program effects Excluded from meta-regression with gender as moderator since % of males was not reported	Outcome measures: DBP: TRF, aggression scale (T) ADHD: n.a. ODD/CD: TRF, aggression scale (T) On-task behavior: n.a.	2
Randomization level	Sample inclusion and exclusion criteria	Age range / grade range at T_0_	Intervention duration and elements of treatment and control arm	Parameters used for effect size calculation and other relevant statistical information	Outcome measures and instruments used for effect size calculation	Study quality^a^
Study *9*. *Prevent-Teach-Reinforce Tertiary Intervention* [60].						
Randomization at individual level, after being matched on age and language ability	*N* = 245 200 boys; 45 girls At risk for EBD based on teacher rating (SSBD), persistence > 6 months, and school absence < 1 time per week on average Only 1 student per teacher could participate, and student with most severe DBP was included	Kindergarten—Grade 8 (7% middle schoolers)	71 days Treatment arm: BP, CB, social skills, curricular modifications, and peer tutoring Control arm: care as usual	Parameters: *M*, *SD* and *N* for T_0_ (baseline) and T_1_ (10 weeks) Baseline differences: race: treatment > control Special remarks: SSRS-PB was not used as measure of DBP or ODD/CD since one third of these items focus on internalizing problems	Outcome measures: DBP: n.a. ADHD: n.a. ODD/CD: n.a. On-task behavior: % of AET (during 2 x 15 minutes)	2
Study *10*. *Good Behavior Game* [61].						
Randomization at classroom level (assigning one classroom within each school to the treatment group and a second classroom in the same school to the control group)	*N* = 570 282 boys; 288 girls Community sample No exclusion criteria	Grade 2–3	2 years Treatment arm: BP (universal program) Control arm: no treatment	Parameters: *M*, *SD* and *N* for T_0_ (baseline) and T_1_ (9 months) Baseline differences: none Special remarks: The exact number of students per group was not reported so authors were contacted in order to obtain this information Analyses did not account for severity of problem behavior	Outcome measures: DBP: Van der Sar’s classroom observation (2004), average of talking and out of seat behavior ADHD: n.a. ODD/CD: n.a. On-task behavior: Van der Sar’s classroom observation, on-task scale	1
Randomization level	Sample inclusion and exclusion criteria	Age range / grade range at T_0_	Intervention duration and elements of treatment and control arm	Parameters used for effect size calculation and other relevant statistical information	Outcome measures and instruments used for effect size calculation	Study quality^a^
Study *11*. [62]						
Randomization at individual level	*N* = 50 42 boys; 8 girls Clinical ADHD based on teacher and parent rating (DSM-IV Rating Scale), symptoms present > 1 year, and onset ≤ 6 year Exclusion criteria: special education, IQ < 80, use of medication, and psychosis or gross neurological, sensory or motor impairment	Grade 3–4	4–5 months Treatment arm: BP and CB Control arm: no treatment	Parameters: *M*, *SD* and *N* for T_0_ (baseline) and T_1_ (5 months) Baseline differences: ADHD-score: treatment > control Special remarks: none	Outcome measures: DBP: CRS, average of ADHD and ODD/CD (T) ADHD: CRS, ADHD scale (T) ODD/CD: CRS, aggression scale (T) On-task behavior: n.r. (only pretest scores were available so effect sizes could not be calculated)	2
Study *12*. *Summer Treatment Program* [63].						
Randomization at individual level	*N* = 260 208 boys; 52 girls Clinical ADHD based on parent interview and parent and teacher ratings Exclusion criteria: IQ < 80, psychosis, medical illnesses psychiatric/neurological disorder requiring separate treatment, and medication intolerance	7–9.9 years; Grade 1–4	14 months 3 treatment arms: Behavior group received BP, CB, social skills, and sport skills; Medication group used methylphenidate; Combined group received behavior and medication treatment. Multimodal: parent and child component Control arm: care as usual	Parameters: *M*, *SD* and *N* for T_0_ (baseline) and T_1_ (14 months) Baseline differences: age: control > treatment Special remarks: Since *N* differed at T_0_ and T_1_, the average *N* was used to calculate ES Only data of behavioral treatment and comparison group were used in this meta-analysis	Outcome measures: DBP: SNAP (T) and COC (observation), average HI and ODD scale ADHD: SNAP (T), average HI and I scale ODD/CD: SNAP (T), ODD scale On-task behavior: n.a.	3
Randomization level	Sample inclusion and exclusion criteria	Age range / grade range at T_0_	Intervention duration and elements of treatment and control arm	Parameters used for effect size calculation and other relevant statistical information	Outcome measures and instruments used for effect size calculation	Study quality^a^
Study *13*. *Modified version of Barkley's program* [64].						
Randomization at individual level	*N* = 92 77 boys; 15 girls Mainly clinical ADHD: 93% were diagnosed by a psychiatrist, the remainder of the sample was in the process of being diagnosed Exclusion criteria: mental retardation	7–10 years	10 weeks Treatment arm: BP and CB; multimodal: also parent component Control arm: waitlist control group with care as usual during assessment period	Parameters: *M*, *SD* and *N* for T_0_ (baseline) and T_1_ (10 weeks) Baseline differences: none Special remarks: none	Outcome measures: DBP: average ADHD (ADHD Rating Scale) and ODD/CD (DSM-IV criteria) (T) ADHD: ADHD Rating Scale (T) ODD/CD: Rating based on eight DSM-IV criteria (T) On-task behavior: n.a.	2
Study *14*. [65]						
Randomization at school level	*N* = 117 90 boys; 27 girls Mainly clinical EBD (85%), based on referral by teachers for externalizing problems No exclusion criteria	Kindergarten—Grade 6	1 year Treatment arm: BP; multimodal: also parent and child components Control arm: waitlist group not receiving intervention during assessment period	Parameters: *M*, *SD* and *N* for T_0_ (baseline) and T_1_ (6 months) Baseline differences: Age: treatment > control ADHD at T_0_: treatment > control Special remarks: none	Outcome measures: DBP: DBD Rating Scale, average ADHD and ODD/CD ADHD: DBD Rating Scale, average HI and I scale (T) ODD/CD: DBD Rating Scale, average ODD and CD scale (T) On-task: n.a.	2
Randomization level	Sample inclusion and exclusion criteria	Age range / grade range at T_0_	Intervention duration and elements of treatment and control arm	Parameters used for effect size calculation and other relevant statistical information	Outcome measures and instruments used for effect size calculation	Study quality^a^
Study *15*. *First Step to Success* [66].						
Randomization at school level	*N* = 286 220 boys; 66 girls At-risk for EBD based on teacher rating (SSBD) No exclusion criteria	Grade 1–3	8 weeks Treatment arm: BP, CB and peer tutoring; multimodal: also parent component Control arm: waitlist group not receiving intervention during assessment period	Parameters: *M*, *SD* and *N* for T_0_ (baseline) and T_1_ (3 months) Baseline differences: SSBI-MBI score at T_0_:Treatment > control Special remarks: SSRS-PB was not used as measure of DBP or ODD/CD since one third of these items focus on internalizing problems	Outcome measures: DBP: SSBD-MBI (T) ADHD: n.a. ODD/CD: n.a. On-task: % of AET (during 2 x 15 minutes)	2
Study *16*. *First Step to Success Early Intervention* [67].						
Randomization at student level	*N* = 200 102 boys; 98 girls At-risk for EBD based on teacher rating (SSBD) No exclusion criteria	Grade 1–3	8 weeks Treatment arm: BP, CB and peer tutoring; multimodal: also parent component Control arm: care as usual	Parameters: *M*, *SD* and *N* for T_0_ (baseline) and T_1_ (3 months) Baseline differences: none Special remarks: SSRS-PB was not used as measure of DBP or ODD/CD since one third of these items focus on internalizing problems	Outcome measures: DBP: SSBD-MBI (T) ADHD: n.a. ODD/CD: n.a. On-task: % of AET (during 2 x 15 minutes)	3
Randomization level	Sample inclusion and exclusion criteria	Age range / grade range at T_0_	Intervention duration and elements of treatment and control arm	Parameters used for effect size calculation and other relevant statistical information	Outcome measures and instruments used for effect size calculation	Study quality^a^
Study *17*. *Behavior Education Support and Treatment* [68].						
Randomization at school level	*N* = 64 Gender ratio n.r. At-risk for EBD based on teacher ratings No exclusion criteria	5–12 years; Grade 1–6	1 year 3 treatment arms: schoolwide (universal program for all students and individual program for non-responders), target school (only for children nominated by teacher), and target home. All involved BP, problem solving, and academic skills Control arm: no treatment control group	Parameters: *M*, *SD* and *N* for T_0_ (baseline) and T_1_ (approximately 8 months) Baseline differences: Age: control > treatment Special remarks: Data of the ‘Target School’-group was used as treatment group Since *N* differed at T_0_ and T_1_, the average *N* was calculated	Outcome measures: DBP: ADS-IV, average ADHD and ODD (T) ADHD: ADS-IV, average HI and I scale (T) ODD/CD: ADS-IV, ODD-scale (T) On-task: n.a.	2
Study *18*. *Electronic Daily Behavior Report Card* [69].						
Randomization at individual level	*N* = 46 37 boys; 9 girls At-risk for EBD based on teachers’ behavior concerns Exclusion criteria: medication use, enrollment in special education program	Grade 1–5	3 weeks 2 treatment arms: daily report card with and without feedback from teacher to parent. Both involved BP Control arm: waitlist group with no intervention during assessment period	Parameters: *M*, *SD* and *N* for T_0_ (baseline) and T_1_ (3 weeks) No baseline differences Special remarks: data of the two treatment arms were averaged and treated as one treatment group	Outcome measures: DBP: TRF, externalizing scale (T), classroom observation (3 x 15 minutes) ADHD: CRS-R, ADHD-scale (T) ODD/CD: n.a. On-task: n.a.	1
Randomization level	Sample inclusion and exclusion criteria	Age range / grade range at T_0_	Intervention duration and elements of treatment and control arm	Parameters used for effect size calculation and other relevant statistical information	Outcome measures and instruments used for effect size calculation	Study quality^a^
Study *19*. *Good Behavior Game* [40].						
Randomization at classroom level	*N* = 758 379 boys; 379 girls Community sample No exclusion criteria	Kindergarten—Grade 2	2 years Treatment arm: BP (universal program) Control arm: no treatment group	Parameters: *M*, *SD* and *N* for T_2_ and T_3_ (9 months) Baseline differences: SES: control < treatment Race: less Dutch/Caucasian children in control group Special remarks: T_2_ and T_3_ were used since extra students were included between T_0_ and T_1_, who differed on nationality and SES from the sample at T_0_. Therefore, the baseline and follow-up assessment from the second year were used (T_2_ and T_3_) Data of male and female were averaged Analyses did not account for severity of problem behavior	Outcome measures: DBP: PBSI (T), externalizing scale consisting of an ODD and CD scale ADHD: n.a. ODD/CD: PBSI (T), externalizing scale On-task: n.a.	2

AET = Academic Engagement Time; ADHD = Attention Deficit Hyperactivity Disorder; ADS-IV = Assessment of Disruptive Symptoms—fourth version; BASC = Behavioral Assessment System for Children; BOSS = Behavioral Observation of Students in School; BP = Behavioral Program; CB = Cognitive-Behavioral elements in intervention; COC = Classroom Observation Code; CRS = Conners’ Rating Scale; CRS-R = Conners’ Rating Scale—Revised; EBD = externalizing behavior disorders (ADHD, ODD, and/or CD); DISC = Diagnostic Interview Schedule for Children; DSM = Diagnostic and Statistical Manual of Mental disorders; DBP = disruptive behavior problem; EBD = Emotional and Behavioral Disorder; GBG = Good Behavior Game; HI = Hyperactivity/Impulsivity scale; I = Inattention scale; IEP = Individualized Education Program; IQ = Intellectual Quotient; ML = treatment program Mastery Learning; n.a. = not assessed; n.r. = not reported; ODD/CD = Oppositional-Defiant Disorder or Conduct Disorder; P = Parent version; PBSI = Problem Behavior at School Interview; RCT = randomized controlled trial; SES = Socioeconomic Status of the child’s family; SNAP = Swanson, Nolan and Pelham Questionnaire; SSBD = Systematic Screening for Behavior Disorders; SSBD-MBI = Systematic Screening for Behavior Disorders—Maladaptive Behavior Index; SSRS-PB = Social Skills Rating System—Problem Behavior; T = Teacher version; T_0_ = first assessment (baseline); T_1_ = second assessment; T_2_, = third assessment (begin 2^nd^ school year); T_3_ = fourth assessment (end 2^nd^ school year); TOCA-C = Teacher Observation of Classroom Adaptation—Checklist; TOCA-R = Teacher Observation of Classroom Adaptation—Revised; TRF = Teacher Report Form.

^a^ Study quality was assessed based on Jadad’s criteria.

^b^ Randomized block design = design in which 2 schools (or in one study 2 school districts) formed a block. Within each block, one school (or school district) was randomly assigned to the treatment arm while the other school was assigned to the control arm. For each study using this design, it is mentioned whether a block referred to schools or school districts, and which criteria schools or school districts were matched on.

**Fig 1:**
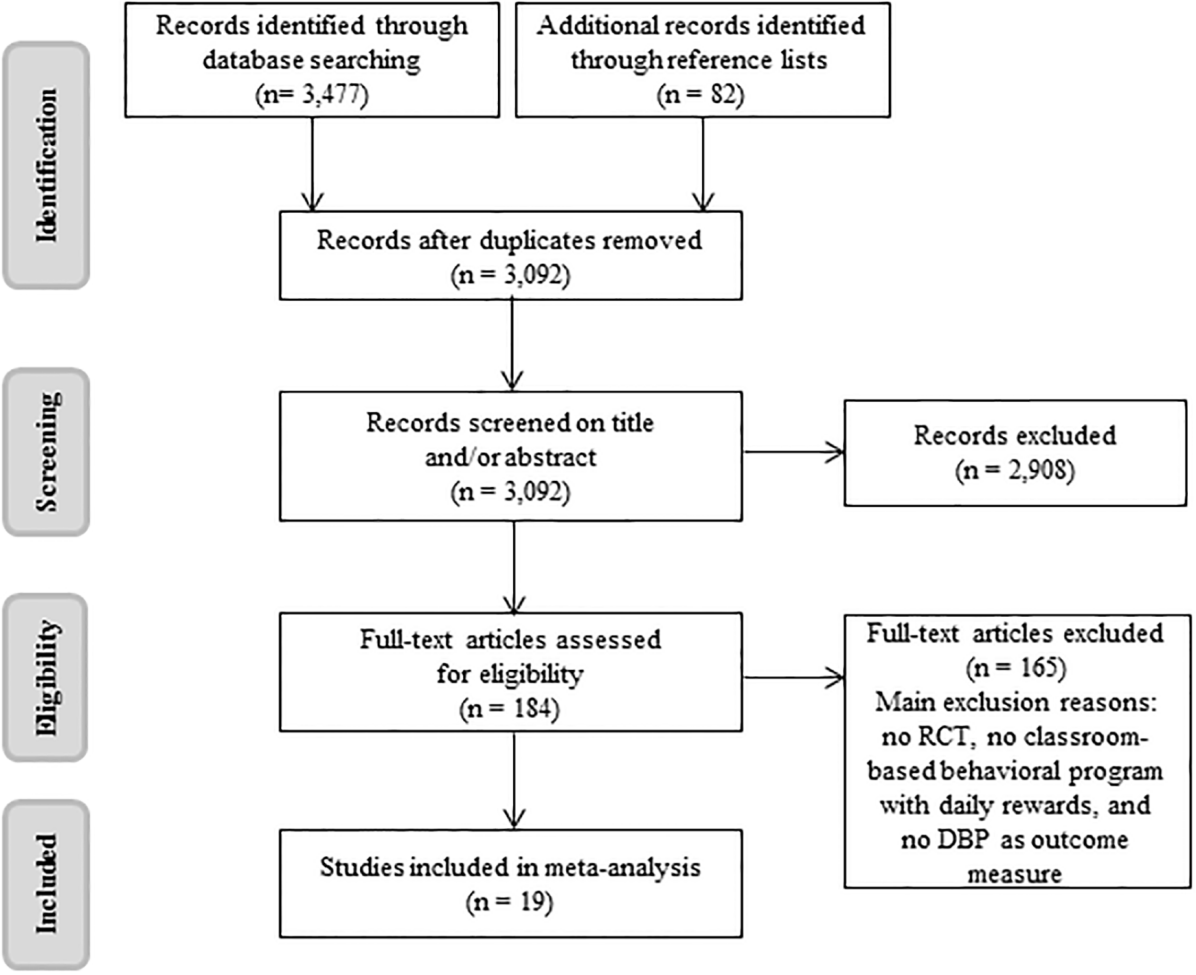
PRISMA flow diagram of studies through the review process.

To identify relevant articles for this meta-analysis, the electronic databases PubMed, Embase, Psycinfo and Eric were searched. Search terms and equivalent mesh terms describing participants’ problem behavior (e.g. ‘disruptive behavior’, ‘externalizing problems’, ‘attention deficit hyperactivity disorder’, ‘oppositional defiant disorder’, ‘conduct disorder’, ‘aggression’) were combined with the term ‘classroom interventions’ and related terms (e.g. ‘teaching method’), which were applied to both titles and abstract (see S2 Supplement for the search terms used in the database PubMed). Reference lists of the selected articles were checked to identify additional relevant studies. To determine eligibility of retrieved articles, we first assessed all inclusion and exclusion criteria on the basis of title and abstract and, if necessary, on the basis of the full-text articles. In case of doubts regarding inclusion and exclusion criteria, authors were contacted to clarify those issues. Eventually 19 articles were included in this meta-analysis. A flow diagram of identification, screening and inclusion of studies is depicted in Fig 1.

### Definitions and outcome measures

Primary analyses focused on disruptive behavior and on classroom-observed on-task behavior. Disruptive behavior was defined as externalizing behavior problems including hyperactivity/impulsivity, oppositional behavior, and aggression (see Table 1 for an overview of the instruments assessing disruptive behavior). For studies reporting several disruptive outcome measures (e.g. ADHD and ODD/CD symptoms), disruptive behavior was measured as a combination of those outcomes by calculating the standardized mean differences and variances for each pertinent subscale and subsequently averaging these scores (assuming a correlation of 1 between the aggregated subscales to yield a conservative estimate [50]). On-task was defined as academic engagement time (AET; mostly measured as the percentage of AET during an interval of 15 minutes; see Table 1).

Post-hoc analyses investigated whether treatment response depended on type of problem behavior (symptoms of ADHD or ODD/CD) or on type of sample (clinical, at-risk, and community). In clinical samples, all participants or a large majority thereof (≥85%) met diagnostic criteria for one or more externalizing behavior disorders. At-risk samples consisted of participants with elevated levels of disruptive behavior problems at school as assessed in each study. In community samples, non-selective samples were studied including entire classrooms or entire schools.

Gender was defined as percentage of male participants in the study sample. Age was defined as the average age (in years) of the study sample at baseline. If age was not reported (in 8 of the 19 studies), an estimation of age was made based on participants’ grade levels (assuming 6.5 years as the average age in grade 1, 7.5 years in grade 2, etcetera [51]), taking into account the percentage of children in each grade. If the percentage of children in each grade was not reported, an equal distribution of children across grades was assumed. Intervention duration was defined in months. Some intervention programs had a duration of more than one year and incorporated multiple post-intervention outcome measurements. For those studies, only the first two measurements were used to maximize homogeneity in intervention duration across studies in the meta-analysis.

### Study quality

The quality of the studies was assessed by two independent researchers (BV and ML) using the Jadad criteria [70] that includes three items of study quality (randomization, double blinding, and withdrawals and dropouts). Each study was scored on a six point scale (0–5), with scores of 2 or less indicating low study quality, and scores of 3 or more points indicating high quality. For all studies, consensus was reached between the researchers.

### Statistical analyses

Statistical analyses were performed using Comprehensive Meta-Analysis [71]. Effect sizes (Cohen’s *d*) were calculated for the effects of the behavioral program within each study for each dependent variable by using the mean, *SD* and sample size of both groups (intervention and control) at baseline and the follow-up assessment. The pooled *SD* was weighted by its inverse variance to control for sample size and measurement error [50]. In order to maximize homogeneity, we used the reported means and *SD*s without any covariates when available. If mean, *SD*, or sample size information were not available, the *t*-value of the effect of the intervention on the dependent variable was calculated by dividing the regression beta coefficient of the group x time interaction by its standard error [72], or the Fisher’s *z* score was calculated using the correlation-value of each dependent variable together with the sample size, which were then converted into Cohen's *d* [50]. For studies reporting results for subgroups (e.g. for males and females separately), the weighted group mean was calculated by multiplying each subgroup mean by its sample size, and then adding the subtotals and dividing the obtained sum by the total sample size [50].

#### Main analyses

First, we investigated meta-analytic effects of behavioral classroom programs on (1) disruptive behavior, distinguishing between teacher-ratings and classroom observations, and on (2) on-task behavior as measured by classroom observations. To test whether the meta-analytic results on these outcome measures were confounded by baseline behavior differences between groups, sensitivity analyses were conducted excluding those studies reporting behavior differences at baseline (the existence of baseline differences are noted in Table 1).

In case of significant intervention effects on teacher-rated disruptive behavior, two additional analyses were performed to ascertain that positive effects were due to behavioral classroom programs rather than interventions in other settings (e.g. parent or child training) or program components other than behavioral techniques (e.g. cognitive behavioral components). One meta-analysis was performed on intervention studies that only involved behavioral components, thus excluding studies into programs that also comprised other treatment components (see Table 1). Another separate meta-analysis tested the specific effect of programs in the classroom setting, by including only studies that were confined to a unimodal teacher program (see Table 1). These two meta-analyses were only conducted on teacher-rated disruptive behavior due to the limited number of studies using classroom observations.

#### Post-hoc analyses

In case of significant intervention effects, several post-hoc analyses were performed to investigate for whom and under which conditions behavioral classroom programs would be most effective. These analyses were only performed on teacher-rated disruptive behavior, as the number of available studies were too limited to carry out these analyses for the other outcome measures. Firstly, program effects were assessed separately for symptoms of ADHD and for ODD/CD to investigate whether effects depended on type of problem behavior. Secondly, the potential moderating effect of severity of problem behavior was investigated by comparing the type of samples (clinical, at-risk and community samples) through a Q-between test, using the nature of the sample as a nominal variable [50]. This analysis investigated whether the efficacy of behavioral programs differed between clinical, at-risk, and community samples. Thirdly, meta-regression analyses were performed to test whether gender (% of male students), age and intervention duration moderated the effects on the outcome measures. There were no missing data on any of the variables, except for one study not reporting the sample’s gender distribution [59]. This study was excluded from the meta-regression analyses on gender.

Given the heterogeneity between trials (e.g. due to differences between the behavioral programs and outcome measures), the random-effects model for heterogeneously distributed data was used to calculate meta-analytic effect sizes [50]. *Q* and *I*_2_ tests were used to test the assumption that the data were heterogeneously distributed. The threshold for performing a meta-analysis was set at the frequently-used limit of three studies [73, 74]. Effect sizes were interpreted applying Cohen’s guidelines, translating *d* = 0.20, 0.50, and 0.80 into thresholds for small, medium, and large effects, respectively [75]. Negative effect sizes indicated that behavioral programs effectively reduced the problem behavior compared to no care or care as usual.

For all meta-analyses, the possibility of publication bias was assessed through Rosenthal’s fail-safe *N* to determine the number of studies necessary to nullify the overall effect and linear regression methods to examine the degree of funnel plot asymmetry [76, 77]. To investigate whether effect sizes were moderated by study quality, meta-regression tested the effect of study quality on the effect sizes obtained for the individual studies. This meta-regression was only performed for teacher-rated disruptive behavior since less than ten studies were available for the other outcome measures, which is required as minimum for adequate meta-regressions [50]. Significance testing was two-sided and *α* was set at 0.05.

## Results

A total of 18,074 children from 19 different studies were included in this meta-analysis. Study characteristics are displayed in Table 1. Seven studies involved children with clinical levels of disruptive behavior (ADHD, ODD or CD), seven studies included children at-risk for externalizing behavior disorders, and five studies used community samples. In the community samples, on average 50% was male (*SD* = 0.58; range 49–53), whereas in studies using clinical or at-risk samples, percentages of male subjects were on average 75% (*SD* = 11.84; range 51–84). While all studies predominantly involved primary school children, seven studies also included children from kindergarten and one study included children from middle school. All studies used behavioral programs as main element of their program, but some studies additionally included cognitive behavioral elements (*n* = 9), social skills training (*n* = 5), peer tutoring (*n* = 5), academic assistance (*n* = 2), problem solving training (*n* = 2), or sport skills training (*n* = 1). Eleven programs were multimodal, of which seven involved a parent training, one involved a child training, and three involved both a parent and child training.

Table 2 provides an overview of all meta-analytic results and heterogeneity statistics. First, the results of the main analyses on disruptive behavior and on-task behavior will be discussed. Thereafter, the post-hoc results will address whether program effects depended on type of problem behavior, type of sample (severity of problem behavior), or on gender, age or intervention duration.

**Table 2:** Overview meta-analytic results of classroom-based behavioral programs on disruptive behavior.

	Meta-analytic effect size						Homogeneity			Publication bias	
	*N*	# of studies	*d*	95% CI		*p*	*Q*	*I*^*2*^	*p*	*p* Egger funnel plot	Fs *N*
Teacher-rated DBP	18,074	17	-0.20	-0.29	-0.10	<0.001	47.36	66.21	<0.001	0.01	176
Observed DBP	907	4	-0.48	-1.11	0.15	0.13	43.33	93.08	<0.001	0.32	13
Observed on-task	1,658	6	0.39	0.21	0.57	<0.001	15.86	68.47	<0.01	0.24	87
Teacher-rated ADHD	13,313	9	-0.19	-0.35	-0.02	0.02	19.92	59.84	0.01	0.03	27
Teacher-rated ODD/CD	16,743	10	-0.15	-0.23	-0.06	<0.01	16.91	46.78	0.05	0.01	51
Sample											
Community	16,138	5	-0.15	-0.30	-0.01	0.04	11.50	73.91	0.01	0.01	22
At-risk	1,081	6	-0.26	-0.42	-0.09	<0.01	18.58	73.00	<0.01	0.36	19
Clinical	828	7	-0.19	-0.35	-0.04	0.01	6.78	11.47	0.34	0.11	9

Negative effect sizes indicate a reduction of disruptive behavior in the treatment condition compared to the control condition.

ADHD = symptoms of Attention Deficit Hyperactivity Disorder; ODD/CD = symptoms of Oppositional Defiant Disorder and/or Conduct Disorder; DBP = Disruptive Behavior Problems; Fs *N* = fail-safe *N*.

### Main analyses

#### Effects of behavioral classroom programs on disruptive behavior

**Fig 2:**
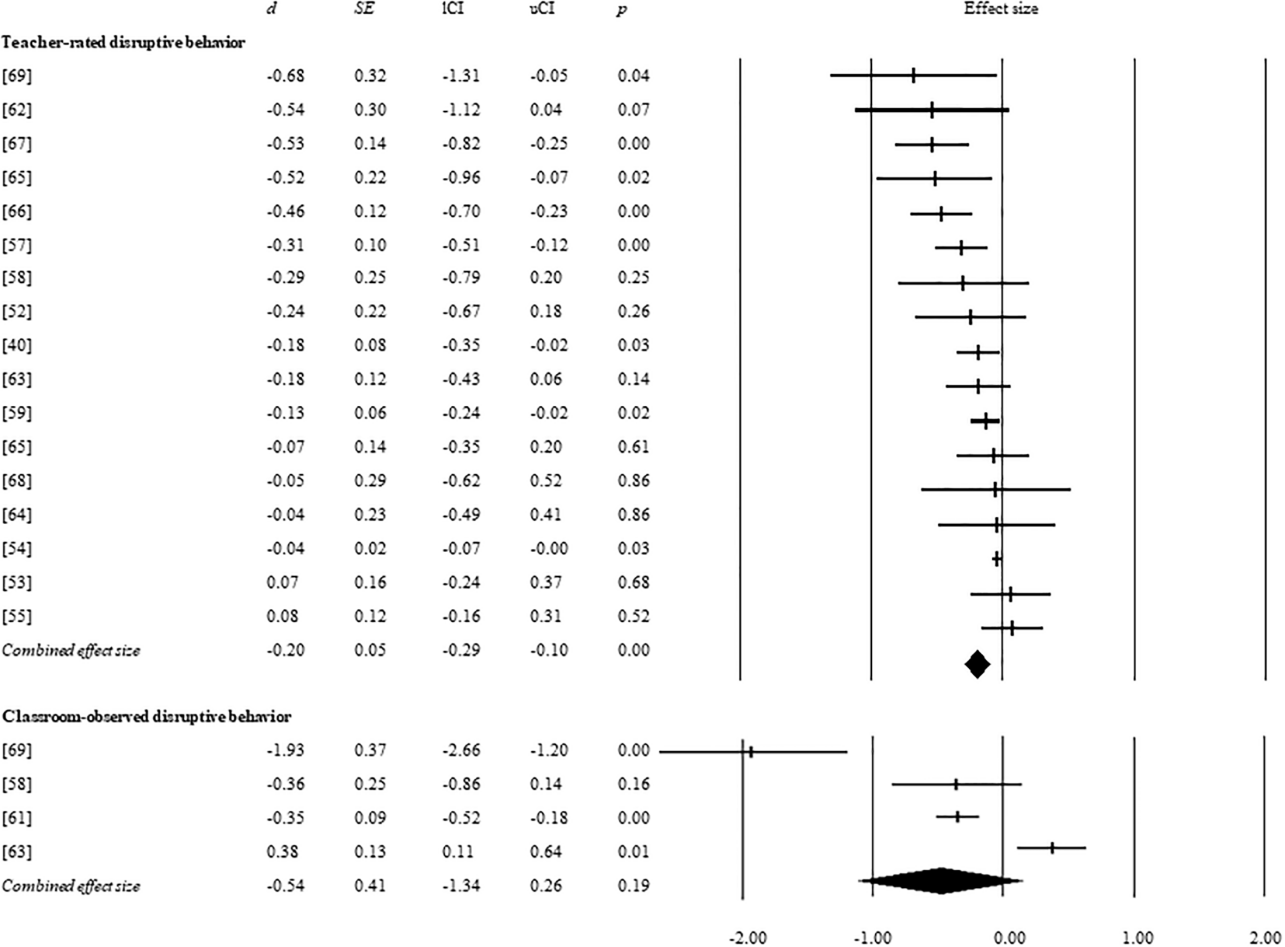
Effect sizes of individual studies and combined effect sizes ondisruptive behavior problems. Meta-analytic results reveal a significant reduction of teacher-rated disruptive behavior in response to behavioral classroom programs.

A total of 17 studies investigated the efficacy of behavioral programs in reducing disruptive behavior through teacher-ratings and three studies used classroom observations by an independent rater as the dependent measure. Results are shown in Fig 2. The meta-analytic results showed that behavioral classroom programs had significant beneficial effects on teacher-rated disruptive behavior with a small effect size (*d* = −0.20, *p* < 0.001), indicating that behavioral classroom programs resulted in larger reductions of disruptive behavior compared to no treatment or treatment as usual. The sensitivity analysis on studies without significant group differences in baseline level of problem behavior revealed a similar significant effect that was somewhat smaller (14 studies, *d* = -0.15, *p* = 0.001). For classroom observations, no significant beneficial effect was found on disruptive behavior (4 studies, *d* = −0.48, *p* = 0.13).

The additional meta-analysis on intervention studies that only involved behavioral management (without other intervention components such as social skills training or peer tutoring) yielded a significant small effect (6 studies, *d* = -0.24, *p* = .01). The separate meta-analysis on the studies that were confined to classroom programs without interventions in other settings (e.g. parent programs), also revealed a significant effect (6 studies, *d* = −0.16, *p* < 0.01). These meta-analytic findings confirm the robustness of the positive effects of behavioral classroom programs on teacher-rated disruptive behavior.

For teacher-rated disruptive behavior, indications of a publication bias were found based on Egger’s regression (*p* = .01), but this bias seemed unlikely based on Rosenthal’s fail-safe *N* statistic which showed that 176 studies were necessary to bring the *p*-value above the alpha-level of .05. There was no evidence of publication bias for classroom-observed disruptive behavior, (*p* = 0.32 based on Egger’s regression and Rosenthal’s fail-safe *N* = 13). Between-study heterogeneity was significant in the meta-analyses on teacher-rated and classroom-observed disruptive behavior, supporting the use of random effects meta-analyses. Study quality was not significantly associated with the studies’ effect sizes for teacher-rated disruptive behavior (*β* = 0.01, *p* = 0.88).

#### Effect of behavioral classroom programs on on-task behavior

Meta-analytic results of six studies using classroom observations to assess on-task behavior, reported a significant beneficial effect that was small in size (*d* = 0.39, *p* < 0.001). A sensitivity analysis on four of these studies that showed no baseline group differences revealed identical findings (*d* = 0.36, *p* = 0.01). There was no evidence of publication bias (*p* = 0.24 based on Egger’s regression and Rosenthal’s fail-safe *N* = 87). Between-study heterogeneity was significant.

### Post-hoc analyses

Additional analyses were conducted on teacher-rated disruptive behavior to investigate which children would benefit most from behavioral classroom programs, and under which circumstances.

#### Program effects on teacher-rated ADHD and ODD/CD symptoms

There were nine studies investigating the effects of behavioral programs on teacher-rated ADHD and nine studies assessing the effects on teacher-rated ODD/CD symptoms. Behavioral programs had significant but small beneficial effects on teacher-rated ADHD symptoms (*d* = −0.19, *p* = 0.02) and teacher-rated ODD/CD symptoms (*d* = −0.15, *p* < 0.01). No indications for publication bias were found for the meta-analytic effect sizes for teacher-rated symptoms of ADHD and ODD/CD based on Rosenthal’s fail-safe *N* (27 and 51 studies, respectively), but Egger’s regression did suggest an asymmetric funnel plot for both outcome measures (*p* = .03 and *p* = .01, respectively). Between-study heterogeneity was significant for teacher-rated ADHD, and close to significant for teacher-rated ODD/CD (*p* = .05).

#### Comparison between clinical, at-risk and community samples

Meta-analytic results on teacher-rated disruptive behavior were calculated for clinical, at-risk and community samples to investigate whether treatment response was stronger for children with higher levels of disruptive behavior compared to children with less problem behavior. Meta-analytic results revealed significant small effects on disruptive behavior in all three samples, although effects were somewhat larger for at risk samples (6 studies, *d* = -0.26, *p* < 0.01), than for clinical and community samples (7 studies, *d* = -0.19, *p* = 0.01 and 4 studies, *d* = -0.15, *p* = 0.04, respectively). The between-group comparison was not significant though (*Q*(2) = 0.95, *p* = 0.62). There was no evidence of publication bias for at-risk (Egger’s *p* = 0.36 and Rosenthal’s fail-safe *N* = 19) and clinical samples (Egger’s *p* = 0.11 and Rosenthal’s fail-safe *N* = 9). For community samples, some evidence of a publication bias was found based on Egger’s regression (*p* = .01), but not based on the Rosenthal’s fail-safe *N* statistic (22 studies). Between-study heterogeneity was significant for the community and at-risk sample, but not for the clinical sample.

#### Moderating effects of intervention duration, age and gender

**Fig 3:**
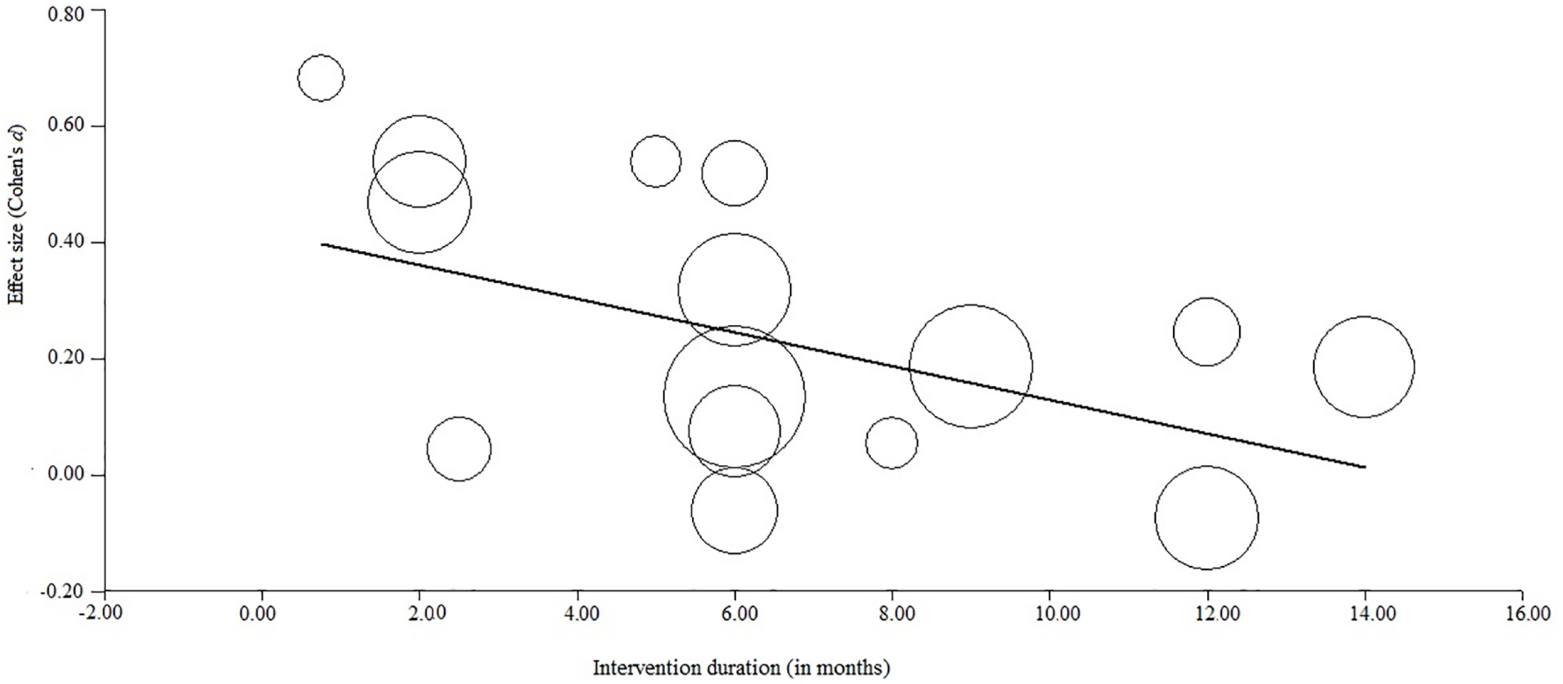
Meta-regression (*N* = 16) revealing a significant negative association between the studies’ effect sizes and intervention duration.

Meta-regression analyses revealed a negative trend between intervention duration and effect sizes for teacher-rated disruptive behavior (*b(SE)* = 0.006(0.003), *p* = 0.06, *R*^2^ = .22), suggesting larger effects for behavioral programs with a shorter duration. To assess the possibility that this trend was driven by one study [54] with a much larger intervention duration (48 months) than the other studies (ranging between 0.8–14 months), the association between effect size and intervention duration was also investigated without that study, now yielding a stronger and significant effect (*b*(*SE*) = -0.03(0.01), *p* = 0.02, *R*^2^ = .43) in the same direction (see Fig 3). Age (ranging between 6–11 years) did not moderate the teacher-rated effect of behavioral programs on disruptive behavior (*b(SE)* = -0.005(0.05), *p* = 0.91). Furthermore, gender (ranging between 49–86% males) did not moderate the teacher-rated effect on disruptive behavior (*b(SE)* = 0.19(0.40), *p* = 0.64), and similar non-significant effects were found when power was increased by inserting the data for boys and girls separately for two studies reporting gender-specific effect sizes [40, 57].

## Discussion

This is the first meta-analysis of RCTs into the efficacy of behavioral classroom programs on elementary school disruptive behavior problems (19 RCT’s, *N* = 18,074 participants). Results confirm our hypothesis that behavioral programs effectively reduce teacher-rated disruptive behavior and improve classroom-observed on-task behavior, albeit with small effects. The effect on classroom-observed disruptive behavior was not significant, but more studies are necessary to confirm these findings given the limited number of studies contributing to this analysis (*n* = 4). Post-hoc results indicated that behavioral classroom programs had similar positive effects on both teacher-rated ADHD symptoms and teacher-rated ODD/CD symptoms. Furthermore, results suggested that program effects were similar for a wide group of children, irrespective of severity of problem behavior, gender, and age. However, programs of a shorter duration were more effective than lengthier programs.

The positive effects that we found for behavioral classroom programs on teacher-rated disruptive behavior are in line with previous reviews and meta-analyses [21, 24, 25, 27]. Those earlier reviews and meta-analyses, however, predominantly included single- or within-subject designs. Our meta-analytic effect sizes are smaller than the effect sizes found in reviews that also included single-subject or within-subject designs [21, 24, 27]. These smaller meta-analytic effect sizes may be explained by our exclusive focus on RCTs in which the risk of bias, and thus of inflated effect sizes, is minimized compared to single-subject or within-subject designs [29–31, 78]. These differences in effect sizes also highlights the importance of RCTs in efficacy trials.

Independent raters not involved in treatment delivery (i.e. less-proximate raters) observed positive effects of behavioral classroom programs. Although these raters did not report positive effects for disruptive behavior, they did report an improvement of on-task behavior. This result implies that children are able to pay more attention to their school work after participating in behavioral classroom programs. Our findings are in line with a recent meta-analysis [32] demonstrating that beneficial effects of behavioral parent interventions (targeting ADHD) can be captured by less-proximate instruments such as behavioral observations. According to the investment argument, teachers involved in treatment delivery may be biased, which could result in inflated effect sizes. Possibly, the lack of an effect on classroom-observed disruptive behavior in the current study, can be explained by the investment argument. However, the present meta-analysis was restricted to three studies and awaits replication with a larger number of studies. We strongly recommend future randomized efficacy trials to use classroom observations as outcome besides most proximate measures such as teacher-ratings, in order to provide a comprehensive view on the efficacy of behavioral programs.

Our results also reveal that behavioral classroom programs were equally effective for clinical, at-risk and community samples, indicating that program effects did not depend on severity of problem behavior. This contrasts with our expectations, because superior program effects were hypothesized for children with clinical levels of disruptive behavior as there would be more room for improvement in these children [37]. Although our results are in line with a meta-analysis where effects of Individualized Positive Behavior Support at school were similar for diagnosed and undiagnosed children [20], other meta-analytic evidence suggests worse effects of psychosocial parent trainings for children with more severe behavior problems [79]. Despite the inconsistent meta-analytic literature regarding the influence of problem severity on treatment effects of psychosocial interventions in general, the hopeful results of this meta-analysis suggest that also children with clinical levels of disruptive behavior problems may benefit from behavioral classroom programs.

The absence of a moderating effect of gender was unexpected [13], because, in general, boys display higher levels of disruptive behavior [80–82] and severity of problem behavior has been associated with higher responsiveness to parent behavior programs due to more room for improvement [37]. Some caution is warranted when interpreting the results of our meta-regressions though, because these analyses can only assess the relationship between the moderator and treatment efficacy *across* trials, and thus are unable to take into account the variability of participant characteristics *within* each study [83]. Individual studies included in the meta-regressions, for example, did reveal a higher treatment response for boys compared to girls [40, 57] and for older compared to younger children [59, 65]. Further, disruptive behavior is less prevalent in girls and effects of interventions are less investigated in girls, making it difficult to draw conclusions on the efficacy of behavioral classroom programs for girls. Since most studies fail to assess moderating effects of gender and age, meta-regressions are necessary to study these moderating effects despite their limitations. Fortunately, variability in age and gender was rather large in our meta-regressions (6.0–10.9 years and 49–84% of male), contributing to the validity of our analyses.

Against our hypothesis, our meta-regression results indicate that behavioral programs with a shorter duration are more effective than more lengthy programs. The unexpected negative association between program duration and effect size might be related to implementation fidelity. Possibly, it was more difficult for teachers to effectively implement more lengthy programs since program implementation often degrades over time and implementation fidelity affects responsiveness to an intervention [84, 85]. Unfortunately, we were unable to assess this hypothesis as almost half of the studies did not report implementation fidelity and the others used many diverse ways to assess implementation fidelity (e.g. teacher reports versus observations, number of elements implemented, quality of implementation, number of days elements were implemented). Future studies should investigate the teachers' implementation fidelity and assess the development of problem behavior over the course of the program to investigate whether implementation fidelity can explain the negative association between program duration and effect size.

Several clinical implications of our results need to be mentioned. First, our results suggest that behavioral classroom programs effectively reduce symptoms of ADHD and ODD/CD for a wide range of children differing in age, gender, and sample. These findings suggest that behavioral classroom programs may be used as universal programs for the entire classroom, so that many children will be able to benefit from these programs at the same time. This approach has an advantage over selective programs targeting individual students, which will, in general, be relatively more time-consuming and thus more expensive. Second, the beneficial program effects found in the current meta-analysis were all small, indicating that other types of treatments (e.g. medication) might be needed to normalize problem behavior of these children in the classroom.

Some limitations have to be taken into account. First, there was heterogeneity between studies as a result of differences between behavioral programs, sample characteristics, as well as type of instruments. However, our results were bolstered by several additional analyses (on studies exclusively focusing on behavioral programs and on studies restricted to unimodal teacher programs) and meta-regression analyses showed that the effects on teacher-rated disruptive behavior were unrelated to age, gender, type of problem behavior or clinical status, highlighting the robustness of our findings. Second, the aggregation of measures of ODD and CD into one outcome measure could be disputed due to, for example, gender- and age-specific differences between these two disorders [81]. Separating these disorders was not feasible though, because we focused on symptoms (of ODD and CD) rather than diagnoses due to the inclusion of community and at-risk samples. Since ODD and CD show a substantial overlap in phenotypical manifestation, separating ODD symptoms from CD symptoms would have been practically impossible [4]. Third, the meta-regression analyses on the moderators age, gender and intervention duration, were only performed on teacher-rated disruptive behavior due to the limited number of studies using parent-ratings or classroom observations. Since many studies included in the meta-regressions involved multimodal programs (e.g. additional parent program) or different components of interventions (e.g. cognitive behavioral elements), findings of the meta-regression might not be specific to behavioral classroom programs. Therefore, future randomized efficacy trials of unimodal behavioral teacher program are necessary to confirm whether these programs are indeed equally effective for a wide group of primary school children.

## Conclusions

Our meta-analysis of 19 RCT studies, including 18,047 elementary school children, showed that behavioral classroom programs result in small but significant improvements of teacher-rated disruptive behavior and classroom-observed on-task behavior at school. Results further suggest that the effects on disruptive behavior are unrelated to age, gender, type of problem behavior (ADHD versus ODD/CD) or clinical status, but that shorter programs are more effective than more lengthy programs. Since the effects of these programs are small, other types of treatments (e.g. medication, or combined psychosocial interventions) or enhancement of treatment fidelity might be needed for normalization of disruptive classroom behavior. Nonetheless, our findings confirm that behavioral classroom programs can contribute to a reduction of disruptive classroom behavior for a large group of children, thus helping to prevent escalation of problem behavior in the classroom.

## Supporting information

S1 Supplement PRISMA checklist. (PDF)

S2 Supplement Search terms used in the database Pubmed. (DOCX)
