# Supplementary material for: Efficacy of behavioral classroom programs in primary school. A meta-analysis focusing on randomized controlled trials
Source: PLoS One. 2018 Oct 10;13(10):e0201779. doi: 10.1371/journal.pone.0201779 (PMC6179198; doi:10.1371/journal.pone.0201779)
Supplement: S2 Supplement — (DOCX) [file pone.0201779.s002.docx]

**S1 Supplement. Search terms used in the database Pubmed**

((((((((((((((((((externalizing problem*[tiab])) OR (externalizing behavi*[tiab])) OR (attention deficit disorder*[tiab])) OR (oppositional defiant disorder*[tiab])) OR ("Attention Deficit and Disruptive Behavior Disorders"[Mesh])) OR (disruptive behavi*[tiab])) OR (aggression*[tiab])) OR (Aggression[Mesh])) OR (conduct disorder*[tiab])) OR ("Conduct Disorder"[Mesh])) OR (attention deficit hyperactivity disorder*[tiab])) OR (adhd[tiab])) OR (Attention Deficit Disorder with Hyperactivity[Mesh])))

AND (((((((((((classroom-based intervention*[tiab])) OR ("Faculty"[Mesh] OR "Schools"[Mesh] OR "Teaching"[Mesh])) OR (primary school*[tiab])) OR (training techni*[tiab])) OR (educational techni*[tiab])) OR (teaching method[tiab] OR teaching methods[tiab])) OR (school*[tiab])) OR (faculties[tiab] OR faculty[tiab])) OR (teaching[tiab] OR teacher[tiab]))))

AND (Child[mh] OR Adolescent[mh] OR pediatrics[mh] OR minors*[tiab] OR boy[tiab] OR boys[tiab] OR boyhood[tiab] OR girl*[tiab] OR kid[tiab] OR kids[tiab] OR child*[tiab] OR adolescen*[tiab] OR juvenil*[tiab] OR youth*[tiab] OR teen*[tiab] OR underage*[tiab] OR under age*[tiab] OR pubescen*[tiab] OR pediatric*[tiab] OR paediatric*[tiab] OR school*[tiab]))) AND

Therapy/Narrow[filter]
